# Supplementary material for: Short Sleep Duration Was Associated with Increased Regional Body Fat in US Adults: The NHANES from 2011 to 2018
Source: Nutrients. 2022 Jul 11;14(14):2840. doi: 10.3390/nu14142840 (PMC9322167; doi:10.3390/nu14142840)
Supplement: Supplementary file 1 [file nutrients-14-02840-s001.zip › nutrients-1783981-supplementary.pdf]

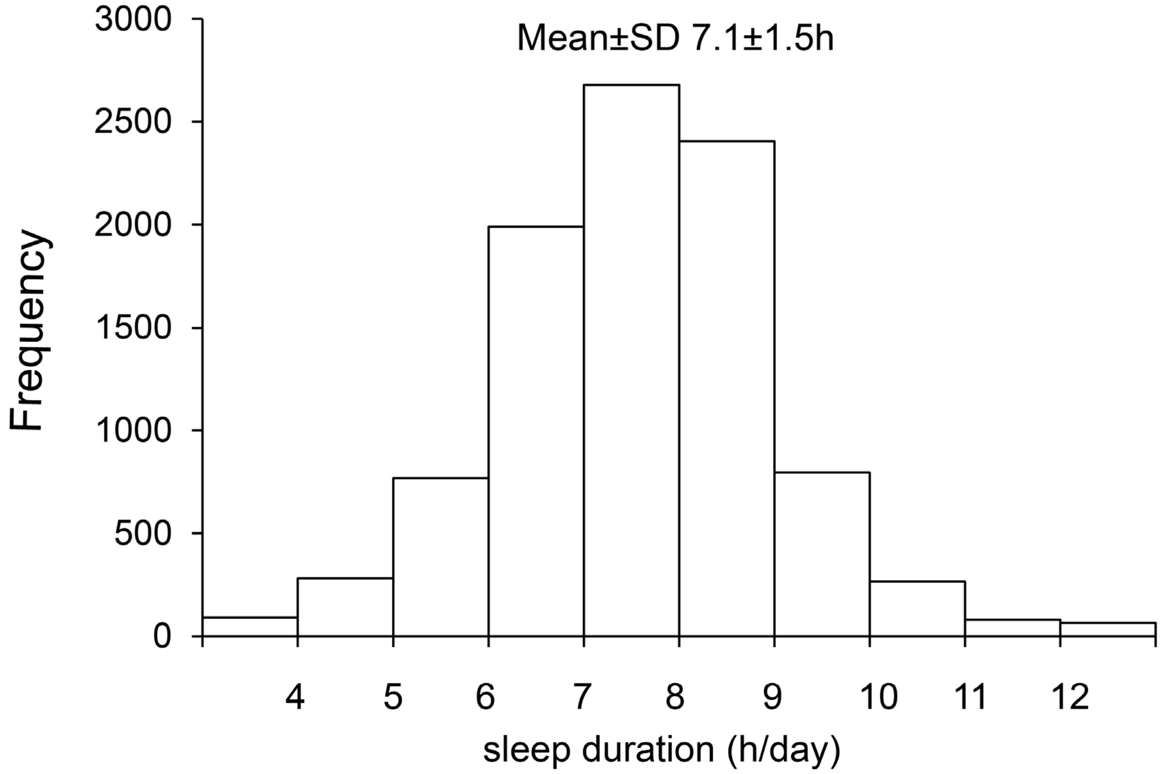

Figure S1. Distribution of sleep duration in the NHANES survey from 2011 to 2018.

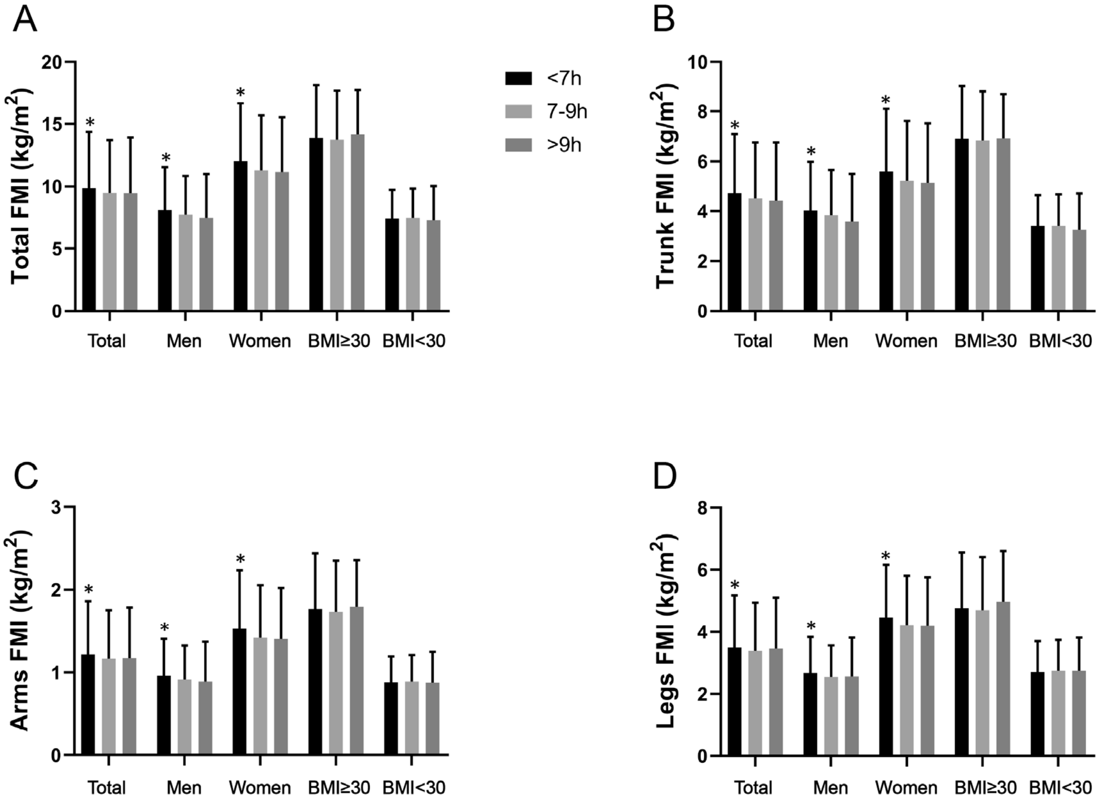

Figure S2. Distribution of different regional fat mass according to short sleep, normal and long sleep duration. Men, n = 4851; Women, n = 4562; BMI ≥ 30, n = 3239; BMI < 30, n = 6174. (A) Total FMI in different groups; (B) Trunk FMI in different groups; (C) Arms FMI in different groups; (D) Legs FMI in different groups. * (higher than normal sleep duration groups) represents $p < 0.01$. FMI: fat mass index.

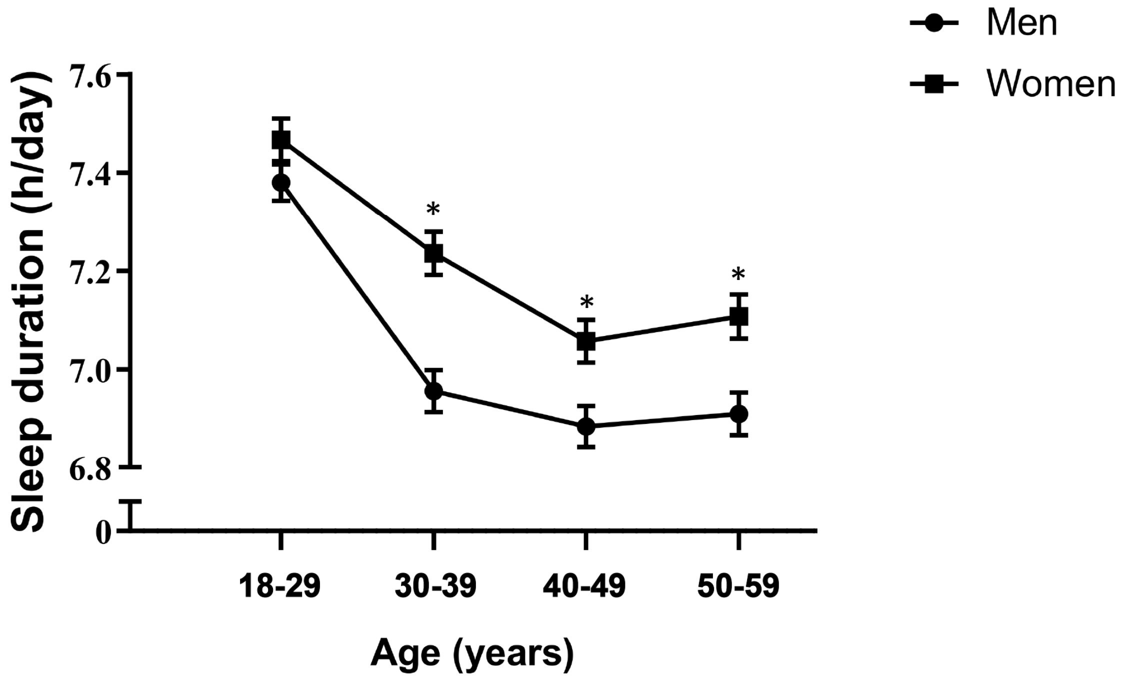

Figure S3. Sleep duration according to age in gender groups. 18–29 years: men (*n* = 1575), women (*n* = 1375); 30–39 years: men (*n*= 1178), women (*n*= 1045); 40–49 years: men (*n*= 1045), women (*n*= 1093); 50–59 years: men (*n*= 1053), women (*n*= 1049). * (higher than men in the same age group) represents $p < 0.01$. Compared to men, women had long sleep duration in 30–39, 40–49, 50–59 years group ($p < 0.01$). In men, there was a decline trend in sleep duration until age 49 ($p < 0.01$). In women, there was a decline trend in sleep duration until age 39 ($p < 0.01$).

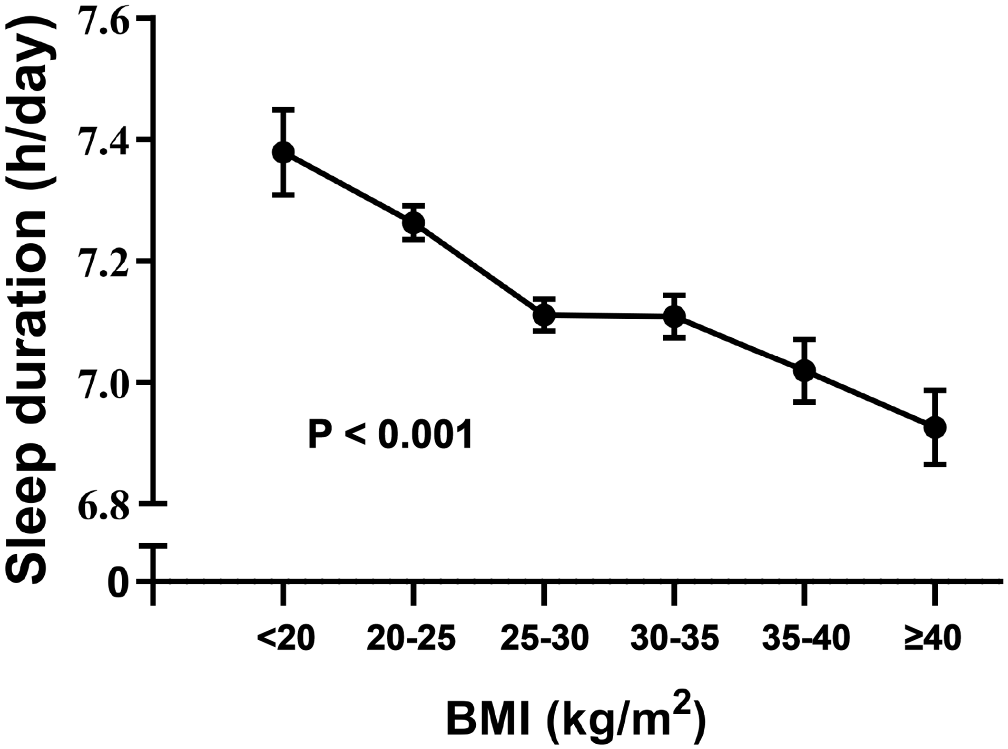

Figure S4. Sleep duration in BMI groups. BMI: <20 (n = 1575); 20–25 (n = 1178); 25–30 (n = 1045); 30–35 (n = 1045); 35–40 (n = 1093); ≥40 (n = 1053). As BMI increased, the average sleep duration decreased ($p < 0.001$). BMI: body fat mass.
